# A retrospective review of vaccine wastage and associated risk factors in the Littoral region of Cameroon during 2016–2017

**DOI:** 10.1186/s12889-022-14328-w

**Published:** 2022-10-23

**Authors:** Rene Nkenyi, Gi Deok Pak, Calvin Tonga, Yun Chon, Se Eun Park, Sunjoo Kang

**Affiliations:** 1 Ministry of Public Health, Littoral Regional Delegation, Douala, Cameroon; 2 International Vaccine Institute, SNU Research Park, 1 Gwanak-ro, Gwanak-gu, Seoul, 06800 Republic of Korea; 3 Ministry of Public Health, Surveillance, Monitoring & Evaluation Section, EPI Central Technical Group, Yaoundé, Cameroon; 4 Yonsei University Graduate School of Public Health, 50-1 Yonsei-ro, Seodaemun-gu, 03722 Seoul, Republic of Korea

**Keywords:** Vaccine wastage, Vaccine coverage, Rural, Urban, Seasonality, Vaccine types, Risk factors, Cameroon

## Abstract

**Background:**

Immunization is an effective preventive health intervention. In Cameroon, the Expanded Program on Immunization (EPI) aims to vaccinate children under 5 years of age for free, but vaccination coverage has consistently remained below the national target. Vaccines are distributed based on the target population size, factoring in wastage norms. However, the vaccine wastage rate (VWR) may differ among various settings. Our study aimed to assess vaccine wastage for different site settings, seasonality, and vaccine types in comparison to vaccination coverage in order to provide comprehensive insights on vaccine wastage.

**Methods:**

A retrospective data collection and analysis were conducted on immunization and vaccine wastage data in the Littoral Region of Cameroon during 2016 and 2017. Health districts were classified as urban or rural, seasonality was categorized as rainy or dry season, and vaccine types were grouped into liquid, lyophilized, oral, and injectable vaccines. VWRs and vaccination coverage rates (VCRs) were calculated, and the vaccine waste factor was investigated.

**Results:**

The VWR of Bacillus Calmette-Guérin (BCG; 32.19%) was the highest, followed by measles and rubella (MR; 19.05%) and yellow fever (YF; 18.34%) among all EPI vaccines in the Littoral Region of Cameroon during 2016 and 2017. Single-dose vaccine vials exhibited lower VWRs than multi-dose vials. Dry season was associated with higher VWRs for most vaccines, although more lyophilized vaccines (BCG, MR, YF vaccines) were wasted in rainy season in 2016. The VWR was persistently higher in rural than urban health districts. The months of February and November saw a decrease in VCRs. The study found an overall negative correlation between VCR and VWR.

**Conclusions:**

Multiple factors may cause wastage of EPI vaccines in Cameroon. Vaccination area characteristics, seasonality, types of vaccines such as multi- or single-dose, lyophilized or injectable vaccines are related to VWRs in Littoral Region. Further research on vaccine wastage and vaccination coverage across Cameroon is needed to better understand the socio-behavioral aspect of vaccine in-take that may affect the level of vaccination and vaccine wastage. Public health system strengthening is warranted to adapt more real-time monitoring of the VWR and VCR for each vaccine in the government’s immunization programs.

**Supplementary Information:**

The online version contains supplementary material available at 10.1186/s12889-022-14328-w.

## Background

Immunization is strongly recommended by the global medical community as an effective preventive medicine to protect children and adults against infectious diseases [1, 2]. Although infectious diseases affect all countries, the burden is higher in many low-and middle-income countries (LMICs) where low vaccination coverage remains one of the major barriers against child morbidity and mortality associated with vaccine preventable diseases (VPDs) [3–5]. Multiple factors may contribute to the uptake of vaccines and vaccination coverage including but not limited to the following: the availability of and access to vaccines; attitudes, perception, and health-seeking behavior towards vaccination by local populations; proper design and management of vaccination programs; appropriate administration of vaccines and vaccine types; vaccination target area characteristics such as urban and rural settings [2]; seasonality; and financial resources and capacity required for the execution and monitoring of immunization programs [6–8]. Further, global vaccine prices may have budgetary and programmatic implications on new vaccine introductions in resource constraint countries, which may hinder vaccination coverage as an increased cost of vaccines adds a financial burden to the local medical care and health system [6, 9–11]. While a comprehensive analysis of such factors affecting vaccination coverage is needed for different settings and countries, a review on vaccine wastage and its causes, challenges, and compromising effect on vaccination coverage could provide some insights on recommendations to reduce missed opportunities for vaccination [6].

According to the World Health Organization (WHO) report in 1997, nearly 43% of vaccines delivered to the developing countries were wasted, largely due to poor infrastructure [10, 12]. Aggregated national statistics showed disparities in vaccine wastage at the local level such as rural and urban settings [13], which were inextricably associated with challenges of infrastructure capacity. Other factors such as poor monitoring and tracking of vaccination programs [14], parents’ reluctance towards vaccination, concerns about vaccine safety, accessibility of health facilities especially in hard-to-reach communities, waiting time at health facilities, low educational level of the local population including both residents and health workers, population density, and logistical challenges in conducting vaccination programs also contributed to vaccine wastage in both rural and urban settings [15–17].

In Cameroon, the Expanded Program on Immunization (EPI) began in 1976 as a coordinated pilot project of the Organization of Coordination for the Control of Endemic Diseases in Central Africa and became operational nationwide in 1982 [18]. The national EPI aims to prevent, control, and eradicate VPDs. Following the Declaration of the Reorientation of Primary Health Care in 1993, the EPI activities were integrated into the Minimum Package of Activities of health facilities nationwide, and the EPI vaccines were given to children free of charge, considering vaccination as a fundamental right of a child [18]. Although the immunization coverage of the EPI vaccines in Cameroon has gradually increased over the past decades, it still falls short of the national target, and there is sufficient evidence of missed or incomplete vaccination of eligible children [19]. Several reasons may explain this trend including the acceptance and uptake of national EPI programs by the general population, as well as challenges related to vaccine logistics and the management of vaccination programs [20] that aimed to not only increase the overall national vaccination coverage but also reduce vaccine wastage [21]. Vaccine wastage has a direct impact on immunization coverage as it translates to the availability of vaccines for use, especially in areas with poor access to vaccine storage facilities [6, 7]. Even when access to vaccine storage facilities is guaranteed, high vaccine wastage increases the cost of immunization programs because vaccine waste factors need to be considered when forecasting and planning the total number of vaccine doses required in each vaccination programs. In this context, reducing vaccine wastage to acceptable levels has been one of the measures recommended by the government of Cameroon to improve the national EPI vaccination coverage (Supplementary Table 1) [18].

The national EPI programs consider the population size of each targeted vaccine to estimate the total number of respective vaccine doses required as well as any potential vaccine wastage that may occur during the implementation phase of vaccinations. Routine monitoring of the vaccine wastage rate (VWR) of each EPI vaccine and utilization of field data for estimating needed vaccine doses are critical for appropriate management of vaccines for immunization programs; they also help avoid or reduce any missed opportunities of vaccination due to vaccine wastage. In this study, we aimed to investigate the VWR of EPI vaccines in the Littoral Region of Cameroon, including by analyzing risk factors such as type of vaccine, seasonality, and characteristics of vaccination sites, in comparison to the vaccination coverage rate (VCR) of respective vaccines. Our study findings may contribute to better understanding the factors causing vaccine wastage in Cameroon, proposing recommendations to improve the management of vaccines and planning, execution, and monitoring of immunization programs, and ultimately enhancing the national EPI coverage.

## Methods

### Study design and inclusion criteria

A retrospective data analysis of the Cameroon government’s immunization records of children under 5 years of age from all 24 health districts in the Littoral Region was conducted, using the District Vaccination Data Management Tool (DVDMT) accessed from the Ministry of Health (MOH). The dataset covered the period from January 1, 2016 to December 31, 2017. The vaccines targeted for our analyses were the bacillus Calmette-Guérin vaccine (BCG); oral polio vaccine (OPV); inactivated polio vaccine (IPV); pentavalent vaccine (PENTA), which included the diphtheria, pertussis, and tetanus (DPT), hepatitis B (HepB), and Haemophilus influenza type b (Hib) vaccines; pneumococcal conjugate vaccine (PCV); rotavirus vaccine (ROTA); measles-rubella vaccine (MR); and yellow fever vaccine (YF). Records of the anti-tetanus vaccine and human papillomavirus (HPV) vaccine were excluded from the study as they are not given to children under 5 years of age.

### Study setting

The Littoral Region is one of the most densely populated regions of Cameroon, with an estimated total population of 3.4 million and a surface area of 20,248 km^2^ [22]. Of the total 189 health districts in Cameroon, 24 are in the Littoral Region. These 24 health districts comprise 3 urban, 9 semi-urban, and 12 rural health districts [23]. Health districts were classified as rural or urban based on their geographical remoteness. Seasonal patterns were characterized as rainy and dry seasons, covering the months from June to November and from December to May, respectively [24]. The rainy season is typically associated with poor accessibility to healthcare facilities due to deteriorating road conditions and frequent power failures, especially in rural districts.

### Data collection and analysis

The dataset covering the Littoral Region in 2016 and 2017 was extracted from the government immunization records, District Vaccination Data Management Tool (DVDMT), based on the authorization obtained from the Ministry of Public Health (MOPH), government of Cameroon. The data collected includes the number of children vaccinated, number of doses received, in-stock, remaining, used, and wasted, types of vaccines (liquid or lyophilized vaccines; single-dose or multi-dose vaccines), route of vaccine administration (oral or injectable vaccines), seasonality (rainy and dry season), and setting (urban and rural) (Table 1). The collected data were entered into an Excel-based spreadsheet and analyzed using R version 3.6.0. The number of children vaccinated and vaccine doses used were compared using the chi-square test of independence to investigate if the expected number of children vaccinated with the doses of vaccines used was significantly different from the observed. The VCR and VWR were calculated using a set of formulas outlined in Table 2 [25].

**Table 1:** Variables used for analyses

Variables			Specifications	Remark
**Dependent**	Children vaccinated		Total number of children vaccinated per vaccine	Used to calculate Vaccine Wastage Rate
	Vaccine doses	Doses Received	Doses received by the health district during the month	
		Doses in stock	Doses in the health district at the beginning of each month (Left-over doses from the previous month)	
		Doses remaining (in sealed vials and not expired)	Doses left in the health district at the end of the month	
		Doses used	Calculated from doses received, doses at the beginning and doses remaining	
		Doses wasted	Calculated as difference between number of children vaccinated and doses used	
**Independent**	Seasons	Dry season	From December to May	Favorable conditions
		Rainy season	From June to November	Unfavorable conditions
	Setting	Rural Areas (12 HD^a^)	Poor road networks and electricity supply	Unfavorable
		Urban Areas (12 HD)	Constant power supply and good road networks	Favorable
	Vaccines categories	Liquid	Oral polio vaccine	Wastage relatively easily managed through the Multi-Dose Vial Policy
			PENTA (DTP-HepB Hib) vaccine	
			Pneumococcal conjugate vaccine	
			Inactivated polio vaccine	
			Rotavirus vaccine	
		Lyophilized	Bacillus Calmette-Guérin vaccine	Potential for conflict between reduction in vaccine wastage and Missed Opportunity to Vaccinate
			Measles and Rubella vaccine	
			Yellow fever vaccine	
		Oral vaccines	Oral polio vaccine	Easily administered
			Rotavirus vaccine	
		Injectable vaccines	PENTA (DTP-HepB Hib) vaccine	Not easily administered (liable to dose estimation and reconstitution errors)
			Pneumococcal conjugate vaccine	
			Inactivated polio vaccine	
			Bacillus Calmette-Guérin vaccine	
			Measles and rubella vaccine	
			Yellow fever vaccine	

^a^ *HD* health district

**Table 2:** Indictors and formula to calculate vaccine coverage and wastage rates

Indicator	Formulae
Vaccination coverage rate	$\frac{Number\ of\ children\ vaccinated}{Number\ of\ eligible\ children}\times 100$
Number of doses used	*Doses received* + *Doses in stock* − *usable doses remaining*
Number of doses wasted	*Doses used* − *Children vaccinated*
Vaccine usage rate	$\frac{Children\ vaccinated}{Doses\ used}\times 100$
Vaccine Wastage Rate (VWR)	$100- Vaccine\ usage\ rate=\frac{Doses\ wasted}{Doses\ used}\times 100$
Vaccine Wastage Factor (VWF)	$\frac{100}{100- Vaccine\ wastage\ rate}=\frac{100}{Vaccine\ usage\ rate}$

## Results

### Vaccine wastage and vaccination coverage rates

**Fig. 1:**
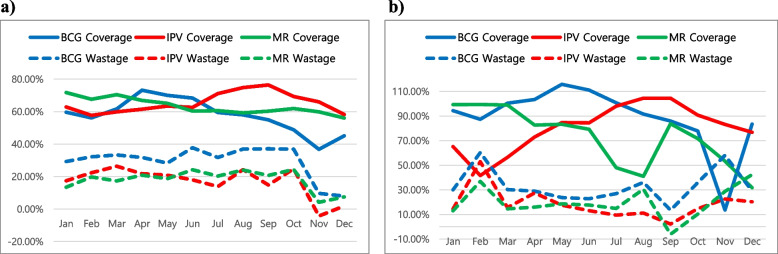
Relationship between vaccination coverage and vaccine wastage for BCG, IPV, and MR in 2016 (**a**) and 2017 (**b**). This figure represents the relationship between vaccination coverage and vaccine wastage rates for BCG, IPV, and MR in the Littoral Region of Cameroon during 2016 (**a**) and 2017 (**b**). The lines in blue, red, and green represent vaccination coverage of BCG, IPV, and MR, respectively. Dotted lines show wastage rates for each vaccine. The y-axis shows the vaccine wastage and vaccination coverage rates in percentages. The x-axis shows the monthly breakdown of 2016 and 2017

During the two-year period of 2016 and 2017, 2640,07 children were vaccinated with the EPI vaccines while 2,851,527 doses were reportedly used, resulting in around 7.42% vaccine wastage. The VWR stratified by each vaccine during 2016 and 2017 exhibited the highest VWR in BCG (number of children vaccinated/number of doses used [percentage]: 172,997/255,125 [32.19%]), followed by MR (148,175/183,042 [19.05%]), YF (153,965/188,533 [18.34%]), and IPV (157,656/191,950 [17.87%]) (Table 3). The single-dose vial vaccines, such as PCV and ROTA, exhibited a negative VWR throughout 2016 and 2017. Overall, the vaccine waste patterns in the investigated vaccines remained similar between 2016 and 2017. A comparative analysis of VWRs and VCRs showed a negative correlation for most vaccines (Fig. 1). The VWR increased each time the VCR decreased, except in 2016 between October and November, during which both vaccination coverage and vaccine wastage decreased simultaneously. In both 2016 and 2017, the vaccination coverage of three vaccines—BCG, IPV, and MR—started high in January but fell immediately in February before increasing again in the following months. Notably, vaccination coverage declined sharply in October and November for all three vaccines, but especially for BCG immunization in both years, although its coverage rate increased again in December.

**Table 3:** Wastage rates and factors for different vaccines in the Littoral Region in 2016 and 2017^a^

Vaccines^**b**^	2016				2017				Total			
	Children vaccinated	Doses used	WR^**c**^	WF^**d**^	Children vaccinated	Doses used	WR	WF	Children vaccinated	Doses used	WR	WF
**BCG**	88,041	128,233	31.34%	1.0031	84,956	126,892	33.05%	1.0033	172,997	255,125	32.19%	1.0032
**OPV**	347,083	360,238	3.65%	1.0004	327,576	344,233	4.84%	1.0005	674,659	704,471	4.23%	1.0004
**IPV**	84,196	102,329	17.72%	1.0018	73,460	89,621	18.03%	1.0018	157,656	191,950	17.87%	1.0018
**PENTA**	259,277	265,547	2.36%	1.0002	241,162	253,707	4.94%	1.0005	500,439	519,254	3.62%	1.0004
**PCV**	259,079	251,142	−3.16%	0.9997	242,642	233,048	−4.12%	0.9996	501,721	484,190	−3.62%	0.9996
**ROTA**	168,835	165,226	−2.18%	0.9998	161,630	159,736	−1.19%	0.9999	330,465	324,962	−1.69%	0.9998
**MR**	81,642	100,052	18.40%	1.0018	66,533	82,990	19.83%	1.0020	148,175	183,042	19.05%	1.0019
**YF**	81,523	99,389	17.98%	1.0018	72,442	89,144	18.74%	1.0019	153,965	188,533	18.34%	1.0018
**Total**	1,369,676	1,472,156	6.96%	1.0007	1,270,401	1,379,371	7.90%	1.0008	2640,077	2,851,527	7.42%	1.0007

^a^ Data source: Cameroon Ministry of Public Health (MOPH), District Vaccination Data Management Tool (DVDMT) 2016–2017 for Littoral Region ^b^ Vaccines: *BCG* bacillus Calmette-Guérin, *OPV* oral polio vaccine, *IPV* inactivated polio vaccine, *PENTA* pentavalent vaccine: diphtheria, pertussis, tetanus (DPT), hepatitis B and Haemophilus influenza type b (Hib) vaccines, *PCV* pneumococcal conjugate vaccine, *ROTA* rotavirus vaccine, *MR* measles and rubella vaccine, *YF* yellow fever vaccine

^c^ *WR* Wastage rate

^d^ *WF* Wastage Factor

### Vaccine wastage per vaccination area and vaccine type

**Fig. 2:**
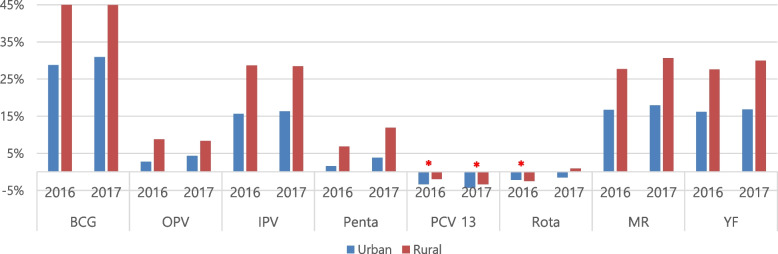
Vaccine wastage comparing rural and urban health districts in 2016 and 2017. Vaccine wastage rates (VWRs, y-axis) in urban and rural health districts are shown as blue and red bars, respectively. Significant differences in VWRs were observed between urban and rural areas for all vaccines in both 2016 and 2017, except for the single-dose PCV and ROTA, with statistically insignificant findings (marked in red asterisk (*))

**Fig. 3:**
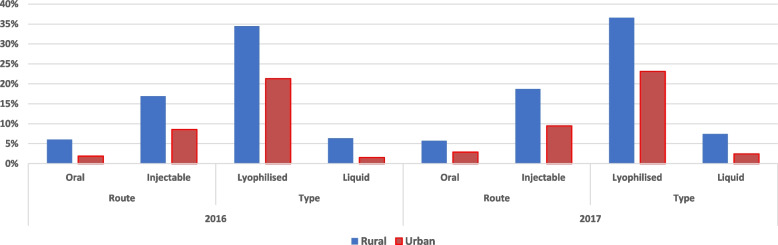
Vaccine wastage rates for different categories of vaccines comparing rural and urban health districts. This figure presents a comparative analysis of the VWRs (y-axis) in rural and urban health districts represented as blue and red bars, respectively. Vaccines investigated are categorized according to the route of administration, such as oral (OPV and rotavirus vaccine) or injectable (PCV, PENTA, BCG, IPV, MR, and YF) and types of vaccines such as lyophilized (BCG, MR, and YF) or liquid (OPV, IPV, PENTA, PCV, ROTA)

The VWR of EPI vaccines analyzed was higher in rural areas than urban areas in both 2016 and 2017, irrespective of the type of vaccine such as the route of administration and form of preservation (Fig. 2). This difference in vaccine wastage was significant: overall VWR of 5.92% (1,177,291 children vaccinated while 1,251,309 vaccine doses used) and 6.89% (1,107,140 children vaccinated; 1,189,029 vaccine doses used) in urban areas compared to 12.89% (192,385 children vaccinated; 220,847 vaccine doses used) and 14.23% (163,261 children vaccinated; 190,342 vaccine doses used) in rural areas in 2016 and 2017, respectively (Table 4). Notably, the lyophilized vaccines (Table 1)— BCG, MR, and YF vaccines—exhibited higher vaccine wastage in both rural and urban health districts (over 15 and 16% wastage in urban areas in 2016 and 2017; over 27 and 29% wastage in rural areas in 2016 and 2017) compared to the other vaccine types. Following the lyophilized vaccines, IPV also showed a high level of vaccine wastage in both urban and rural areas in 2016 and 2017 (Table 4, Fig. 3). The difference in the VWR between urban and rural areas was the highest for BCG, followed by IPV, YF, and MR in 2016. The VWR was higher in rural than urban areas by 16.15%-point, 12.99%-point, 11.38%-point, and 11.00%-point in BCG, IPV, YF, and MR respectively in 2016; and by 13.93%-point, 13.12%-point, 12.74%-point, and 12.15%-point in BCG, YF, MR, and IPV in 2017 (Table 4). These were also injectable vaccines (Table 1), which had higher vaccine wastage than oral vaccines (Table 4).

**Table 4:** Vaccine wastage comparing rural and urban health districts

Vaccines^**a**^	2016								2017							
	Urban			Rural			χ2^**b**^	***p*** value	Urban			Rural			χ2	***p*** value
	Children vaccinated	Doses used	Wastage	Children vaccinated	Doses used	Wastage			Children vaccinated	Doses used	Wastage	Children vaccinated	Doses used	Wastage		
**BCG**	76,912	108,017	28.80%	11,129	20,216	44.95%	410.93	< 0.0001	74,408	107,754	30.95%	10,548	19,138	44.88%	300.01	< 0.0001
**OPV**	298,562	307,052	2.77%	48,521	53,186	8.77%	88.29	< 0.0001	285,696	298,535	4.30%	41,880	45,698	8.35%	35.58	< 0.0001
**IPV**	72,580	86,051	15.65%	11,616	16,278	28.64%	161.70	< 0.0001	64,471	77,052	16.33%	8989	12,569	28.48%	112.35	< 0.0001
**PENTA**	221,968	225,482	1.56%	37,309	40,065	6.88%	50.81	< 0.0001	209,745	218,045	3.81%	31,417	35,662	11.90%	111.64	< 0.0001
**PCV**	221,762	214,541	−3.37%	37,317	36,601	−1.96%	2.97	0.0851*	211,082	202,505	−4.24%	31,560	30,543	−3.33%	1.02	0.3127*
**ROTA**	144,832	141,803	−2.14%	24,003	23,423	−2.48%	0.11	0.7411*	140,414	138,329	−1.51%	21,216	21,407	0.89%	5.26	0.0215
**MR**	70,388	84,489	16.69%	11,254	15,563	27.69%	111.85	< 0.0001	57,970	70,639	17.93%	8563	12,351	30.67%	124.12	< 0.0001
**YF**	70,287	83,874	16.20%	11,236	15,515	27.58%	118.60	< 0.0001	63,354	76,170	16.83%	9088	12,974	29.95%	136.62	< 0.0001
**Total**	1,177,291	1,251,309	5.92%	192,385	220,847	12.89%	521.30	< 0.0001	1,107,140	1,189,029	6.89%	163,261	190,342	14.23%	513.93	< 0.0001

* Statistically insignificant

^a^ Vaccines: *BCG* bacillus Calmette-Guérin, *OPV* oral polio vaccine, *IPV* inactivated polio vaccine, *PENTA* pentavalent vaccine: diphtheria, pertussis, tetanus (DPT), hepatitis B (HepB) and Haemophilus influenza type b (Hib) vaccines, *PCV* pneumococcal conjugate vaccine, *ROTA* rotavirus vaccine, *MR* measles and rubella vaccine, *YF* yellow fever vaccine

^b^ χ2 refers to the chi-square test which brings out statistical differences between number of children vaccinated and doses of vaccines used between urban and rural setting

### Seasonality and vaccine wastage rates per vaccine type

**Fig. 4:**
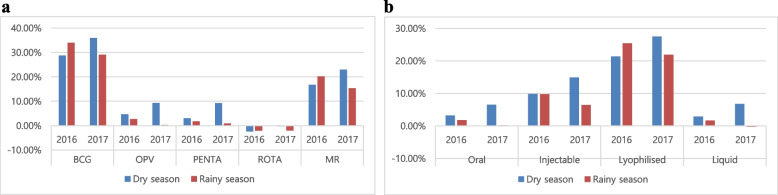
Vaccine wastage rates for various vaccines in dry and rainy seasons. The figure shows different vaccine wastage rates (VWRs) for vaccines and vaccine types in the Littoral Region during 2016 and 2017. The y-axis shows the percentage of the VWR. The x-axis shows: **a** different vaccines; and **b** categories of vaccines, whereby vaccines are grouped by route of administration (oral and injectable) and form (lyophilized and liquid). The blue and red bars indicate the VWR for the dry and rainy seasons, respectively

Overall, VWRs were higher in the dry season than in the rainy season: VWR of 7.23% (666,514 children vaccinated; 718,497 vaccine doses used) in dry season compared to 6.70% (703,162 children vaccinated; 753,659 vaccine doses used) in rainy season in 2016; and 11.88% (610,764 children vaccinated; 693,075 vaccine doses used) in dry season compared to 3.88% (659,637 children vaccinated; 686,296 vaccine doses used) in rainy season in 2017 (Table 5). In 2016, comparatively more vaccines were wasted during the dry season in all vaccine categories (Table 1) except for the lyophilized vaccines (BCG, MR, YF); in 2017, higher vaccine wastage in dry season than rainy season was observed in all vaccine categories (Fig. 4, Table 5). In 2016, more lyophilized vaccines were wasted during the rainy season, whereas more liquid vaccines (PENTA, OPV, and IPV) were wasted in the dry season (Table 5). Of all the vaccines, the biggest difference in vaccine wastage occurred in IPV in 2017, with a 25.15% VWR in the dry season, which was 12.99%-point higher than the VWR of 12.16% in rainy season (Table 5). The VWR of all vaccines was significantly different between the rainy and dry seasons (Table 5), except for the single-dose vaccines (ROTA and PCV) in 2016.

**Table 5:** Vaccine wastage rates comparing dry and rainy seasons

Vaccine^**a**^	2016								2017							
	Dry season			Rainy season			χ2^**b**^	***p*** value	Dry season			Rainy season			χ2^**b**^	***p*** value
	Children vaccinated	Doses used	Wastage	Children vaccinated	Doses used	Wastage			Children vaccinated	Doses used	Wastage	Children vaccinated	Doses used	Wastage		
**BCG**	46,528	65,347	28.80%	41,513	62,886	33.99%	74.48	< 0.0001	46,621	72,830	35.99%	38,335	54,062	29.09%	131.16	< 0.0001
**OPV**	169,175	177,408	4.64%	177,908	182,830	2.69%	18.05	< 0.0001	157,827	174,001	9.30%	169,749	170,232	0.28%	376.18	< 0.0001
**IPV**	39,058	48,331	19.19%	45,138	53,998	16.41%	13.11	0.0003	30,332	40,522	25.15%	43,128	49,099	12.16%	252.87	< 0.0001
**DPT-HepB-Hib**	123,095	126,904	3.00%	136,182	138,643	1.78%	5.15	0.0232	111,558	122,948	9.26%	129,604	130,759	0.88%	240.39	< 0.0001
**PCV**	122,782	119,434	−2.80%	136,297	131,708	−3.48%	1.38	0.2402*	111,502	109,070	−2.23%	131,140	123,978	−5.78%	34.36	< 0.0001
**Rotavirus vaccine**	80,297	78,437	− 2.37%	88,538	86,789	−2.02%	0.25	0.6175*	75,031	74,839	−0.26%	86,599	84,897	−2.00%	5.96	0.0147
**MR**	42,743	51,316	16.71%	38,899	48,736	20.18%	20.37	< 0.0001	37,778	49,033	22.95%	28,755	33,957	15.32%	80.28	< 0.0001
**YF**	42,836	51,320	16.53%	38,687	48,069	19.52%	14.79	< 0.0001	40,115	49,832	19.50%	32,327	39,312	17.77%	4.45	0.0349
**Total**	666,514	718,497	7.23%	703,162	753,659	6.70%	5.85	0.01557	610,764	693,075	11.88%	659,637	686,296	3.88%	1245.01	< 0.0001

*Statistically insignificant

^a^Vaccines: *BCG* bacillus Calmette-Guérin, *OPV* oral polio vaccine, *IPV* inactivated polio vaccine, *DPT-HepB-Hib* pentavalent vaccine: diphtheria, pertussis, and tetanus (DPT), hepatitis B (HepB) and Haemophilus influenza type b (Hib) vaccines, *PCV* pneumococcal conjugate vaccine, *MR* measles and rubella vaccines, *YF* yellow fever vaccine

^b^χ2 refers to the chi square test which brings out statistical differences between number of children vaccinated and doses of vaccines used between dry and rainy seasons

## Discussion

To achieve the full effect of immunization, high vaccination coverage and low vaccine wastage are important. High vaccine wastage makes vaccines less available for use, especially in remote areas where access to the central vaccine storage facility is challenging. To avoid compromising public health efforts towards increasing the vaccination coverage of EPI vaccines and minimizing vaccine wastage, an accurate demand-forecasting of these vaccines for target immunization populations and regular monitoring of vaccine wastage at all levels are important. The general guidelines on the VWR per vaccine [26] notes the wastage rates of 50% for BCG and 25% for the measles vaccine are considered acceptable for reconstituted vaccines; 10% for OPV; 15% for liquid vaccines in multi-dose vials of 10 or more doses; and 5% for liquid vaccines in single or two-dose vials such as PENTA and PCV. Considering this standard, the VWRs of each EPI vaccine in Cameroon during 2016 and 2017 were at an acceptable level; the VWR of BCG remained around 31–33%, and the VWRs of OPV and PENTA were below 5%. Country-specific vaccine procurement and management capacities are essential to achieve such VWR targets. In Cameroon, the targeted VWR [21] under the routine EPI during 2016 and 2017 was influenced by the government’s commitment to provide more resources to the EPI program, such as setting up a comprehensive multi-year plan (MINSANTE: Comprehensive multiyear plan 2007–2011 of the expanded program on immunization, unpublished) and supplementary immunization activities in health districts with poor performance indicators.

In the Littoral Region of Cameroon, lyophilized vaccines showed a higher VWR. This finding is similar to that of an existing study from The Gambia [10], which showed higher wastage rates in lyophilized vaccines than in other types of vaccines. In our study, VWRs were lower than the findings from a study in Bangladesh [27], where the wastage rate for BCG was nearly 84.9%, followed by MR at 69.7%, and PENTA at 44.4%. Notably, the liquid vaccine IPV also exhibited a high wastage rate (17.9%) in the Littoral Region, which may be due to its introduction into the Cameroon government’s EPI program in June 2015 [28]. Its high VWR may be attributable to the early stage of vaccine introduction as typically experienced in any new immunization program [29]. Our study supports the existing literature on lower wastage rates for vaccines that follow the multi-dose vial policy (MDVP), as seen in other studies from the Northwest Region of Cameroon and Bangladesh [27].

Using opened MDVP vials within 28 days, provided the storage conditions are favorable [30], is expected to reduce vaccine wastage [31]. However, lyophilized vaccines (BCG, MR, and YF) must be used within 6 h after reconstitution, or at the end of the vaccination session, whichever comes first, after which these vaccines must be discarded irrespective of the doses used in the vial [32]. Hence, vaccine wastage of these vaccines is only avoidable during large-scale vaccination sessions, which last less than six hours. Therefore, lyophilized vaccines tend to have a higher wastage rate than liquid vaccines (OPV, IPV, PENTA, PCV, and ROTA) in the real-world setting.

Understanding the relationship between vaccination coverage and vaccine wastage is a basic starting point to investigating causes and risk factors associated with vaccine wastage. Optimally, if a vaccination program is conducted based on a well-planned immunization plan, such as proper microplanning involving effective community engagement and following the standard operating procedures of appropriate vaccine management, vaccine wastage should remain low and vaccination coverage high. Our study showed an overall negative correlation between vaccination coverage and vaccine wastage and presented the multifaceted risk factors contributing to vaccine wastage. The lower vaccination coverage may not necessarily be solely due to the unavailability of vaccines or high vaccine wastage. Conversely, low vaccination coverage may also cause high vaccine wastage as vaccines in stocks can be damaged at health facilities, resulting in insufficient vaccines to immunize the target populations. This is highly possible as leftover vaccines taken to outreach sites may not return to the cold chain in their optimal conditions [33] and may be discarded. Notably, our study found that in the Littoral Region of Cameroon during October and November 2016, the wastage of all vaccines decreased when vaccination coverage also decreased. This may be due to the lower availability of vaccines or adoption of strategies that helped reduce vaccine wastage but compromised vaccination coverage [6]. The former is the likely explanation in the Littoral Region as BCG was not available even at the central vaccine storage facility in Yaoundé during the study period. The lack of a particular vaccine has a demotivating effect on healthcare workers in organizing vaccination sessions, as they will need to reorganize such sessions when the missing vaccines becomes available. Further, parents are demotivated to come for vaccination if they are aware of frequent vaccine shortages.

Rural areas are characterized by a smaller, sparsely distributed population, resulting in conditions that favor a high VWR [13, 16, 31]. This has been the case in the Littoral Region, where over the 2-year study period, rural districts had higher VWRs. Compared to urban health districts that mostly employ a fixed vaccination strategy (children are brought to health facilities for vaccination), in rural districts, an outreach vaccination strategy is typically applied to reach people living in remote areas with limited access to health facilities [13]. Usually, vaccine vials taken out for this strategy do not return to the vaccine storage facilities if vaccine vial monitors (VVM; small stickers that adhere to vaccine vials and change color as the vaccine is exposed to heat, letting health workers know whether the vaccine can be safely used for immunization) are not in place. Existing studies have reported high VWRs in rural areas due to vial breakage while opening, the burden of cost expenditure, and improper handling and storage, all of which were often related to the lack of skilled personnel in rural immunization activities [7, 9, 16, 33]. Furthermore, the possibility of accidents occurring in rural areas leading to unopened vial breakage is higher than in urban areas. Relatively less skilled personnel may also be involved in rural immunization activities [31]. Not fully understanding the importance of vaccination due to a low educational level, rural populations tend to have negligent behavior toward meeting vaccination appointments [17]. This often leads to waste of open vials, especially lyophilized vaccines. Notably, such differences in rural and urban vaccine wastage were not significant in a study conducted in The Gambia, likely due to enhanced vaccine management and high vaccination coverage [10]. In the Littoral Region of Cameroon, attempts are being made to reduce vaccine wastage in rural areas and nearby health facilities by planning immunization sessions more strategically that included increasing the size of vaccinated target populations.

The two major seasons in Cameroon, dry and rainy, have a distinctively different effect on immunization activities. The rainy season is typically associated with poor accessibility to healthcare facilities due to deteriorating road conditions and frequent power failures, especially in rural districts. This negatively impacts the vaccine supply chain and increases accidents that result in wastage of unopened vaccine vials during the outreach sessions of immunization programs [34]. Typically, during the rainy season, parents are more likely to miss vaccination appointments, which may result in increased vaccine wastage, especially for lyophilized vaccines. This is probably why vaccine wastages for BCG, MR, and YF vaccines were higher during the rainy season than dry season in 2016. Although the dry season is very dusty, it has favorable weather, road, and energy supply conditions. However, in the dry season, ambient temperatures are higher, which may lead to high vaccine wastage if compounded with inadequate cold chain facilities. This likely explains what happened in 2017, when the VWR for all vaccines was higher during the dry season in the Littoral Region.

Our study has also found that some vaccines, particularly PCV and ROTA, exhibited a negative VWR throughout 2016 and 2017. This may be related to poor data quality, which also limits confidence across other findings. However, it may be due to skillful health workers tapping the “extra dose” available in vials, which is due to vaccine manufacturers filling vials with excess volume [35]. Some VWR challenges related to certain vaccines are also closely linked to the respective vaccine cold chain requirement and management. Efforts are underway to develop vaccines that can tolerate extreme temperatures or being out-of-cold chain for a certain period or under monitored and controlled conditions [36]. The vaccines analyzed in this study are not available for controlled temperature chain (CTC) usage, but such CTC could be an innovative approach and contribute to reducing vaccine wastage and enhancing the vaccination coverage of at-risk populations living in rural, remote, or hard-to-reach areas with limited cold chain conditions and infrastructure. Our study also has limitations given that the analyzed data were available secondary data extracted from the government immunization records. The accuracy of the results presented depends on the accuracy of the source data accessed, and more in-depth analysis on the vaccine wastage rates for opened- and closed-vials could not be conducted.

## Conclusions

Investigating vaccine wastage is important to better understand the reasons for VWR and plan for improved immunization programs going forward. To reduce vaccine wastage in the Littoral Region of Cameroon, emphasis should be placed on the risk factors related to rural areas during the rainy season (especially for lyophilized vaccines), and further investigation is needed to understand the causes of high vaccine wastage during the dry season. Improved vaccine cold chain systems should be put in place by investing in basic social infrastructure, such as adequate energy sources for field vaccine storage capabilities. Considering the diverse geographical and climatic characteristics of the Littoral Region, better vaccine demand forecasting with more real-time and site-specific monitoring of VWRs is recommended to prevent the inappropriate supply of vaccines. Further research is needed to more comprehensively analyze vaccine wastage across Cameroon, including by examining socio-behavioral aspects of vaccine acceptance and health-seeking behaviors of local populations to develop more refined public health immunization policy interventions in diverse settings.

## Supplementary Information

**Additional file 1: Supplementary Table 1.** Vaccination coverage and vaccine wastage rate targets in Cameroon in 2017.

## Data Availability

The dataset used in this study are available in the CTG of EPI, MOPH, Cameroon.

## Abbreviations

BCG: Bacillus Calmette-Guérin vaccine
CTC: Controlled temperature chain
CTG: Central Technical Group
DPT: Diphtheria, pertussis, tetanus vaccine
DVDMT: District Vaccination Data Management Tool
EPI: Expanded Program on Immunization
HD: Health district
HepB: Hepatitis B vaccine
Hib: Haemophilus influenza type b vaccine
HPV: Human papillomavirus vaccine
IPV: Inactivated polio vaccine
MOPH: Ministry of Public Health
MR: Measles and rubella vaccine
OPV: Oral polio vaccine
PCV: Pneumococcal conjugate vaccine
PENTA: Pentavalent (DPT-HepB-Hib) vaccine
ROTA: Rotavirus vaccine
VCR: Vaccination coverage rates
VPDs: Vaccine preventable diseases
VWF: Vaccine wastage factor
VWR: Vaccine wastage rate
WF: Wastage factor
WHO: World Health Organization
WR: Wastage rate
YF: Yellow fever vaccine
