# Supplementary material for: A retrospective review of vaccine wastage and associated risk factors in the Littoral region of Cameroon during 2016–2017
Source: BMC Public Health. 2022 Oct 23;22:1956. doi: 10.1186/s12889-022-14328-w (PMC9590201; doi:10.1186/s12889-022-14328-w)
Supplement: Supplementary file 1 — Additional file 1: Supplementary Table 1. Vaccination coverage and vaccine wastage rate targets in Cameroon in 2017. [file 12889_2022_14328_MOESM1_ESM.docx]

**Supplementary Table 1. Vaccination coverage and vaccine wastage rate targets in Cameroon in 2017**

| **Vaccine** | **Targeted coverage (%)^1^** | **Targeted wastage rate (WR) (%)^1^** | **WHO acceptable WR (%)^2^** |
| --- | --- | --- | --- |
| BCG^3^ | 89 | 25 | 50 |
| OPV^4^ | 89 | 10 | 10 |
| IPV^5^ | 79 | 10 | 15 |
| DPT-HepB-Hib 3rd dose^6^ | 89 | 6 | 15 |
| PCV^7^ | 89 | 3 | 5 |
| ROTA^8^ | 82 | 5 | 5 |
| MR^9^ | 89 | 25 | 25 |
| YF^10^ | 89 | 25 | 25 |

^1^ Source of data: Cameroon Ministry of Public Health (MOH), District Vaccination Data Management Tool (DVDMT) 2016–2017 for Littoral Region.

^2^ World Health Organization. (‎2005)‎. Monitoring vaccine wastage at country level: guidelines for programme managers. World Health Organization. Available at: https://apps.who.int/iris/handle/10665/68463

^3^ BCG: bacillus Calmette-Guérin vaccine

^4^ OPV: oral polio vaccine

^5^ IPV: inactivated polio vaccine

^6^ DPT-HepB-Hib (pentavalent): diphtheria, pertussis, and tetanus (DPT), hepatitis B (HepB) and Haemophilus influenza type b (Hib)

^7^ PCV: pneumococcal conjugate vaccine (PCV)

^8^ ROTA: rotavirus vaccine

^9^ MR: measles and rubella vaccine

^10^ YF: yellow fever vaccine
